# A novel highly antifungal compound ZJS-178 targeting myosin I inhibits the endocytosis and mycotoxin biosynthesis of *Fusarium graminearum*

**DOI:** 10.1007/s44297-024-00034-z

**Published:** 2024-09-26

**Authors:** Qiaowan Chen, Chang Liu, Hao Qi, Ningjie Wu, Zunyong Liu, Qin Tian, Xiao-Xiao Zhu, Xiangdong Li, Yun Chen, Zhonghua Ma

**Affiliations:** 1 Institute of Biotechnology, Key Laboratory of Molecular Biology of Crop Pathogens and Insects, Zhejiang University, Hangzhou, 310058 China; 2 Zhejiang Research Institute of Chemical Industry, Hangzhou, 310023 China; 3 State Key Laboratory of Integrated Management of Pest Insects and Rodents, Institute of Zoology, Chinese Academy of Sciences, Beijing, 100101 China; 4 University of Chinese Academy of Sciences, Beijing, 100049 China

**Keywords:** *Fusarium graminearum*, 2-cyanoacrylate compounds, FgMyoI, Endocytosis, Mycotoxin

## Abstract

Chemical fungicides remain a primary tool for managing Fusarium head blight (FHB) due to the lack of resistant wheat cultivars. In this study, we synthesized 101 2-cyanoacrylate compounds and identified ZJS-178 as particularly effective against *Fusarium graminearum* (the causal agent of FHB), inhibiting both hyphal growth and conidial germination. Notably, ZJS-178 is specific to *Fusarium* species, with no impact on other fungi. It also shows no cross-resistance with other groups of fungicides, suggesting a unique mode of action. Mechanistic studies revealed that ZJS-178 targets myosin I in *F. graminearum*, inhibiting ATPase activity and disrupting endocytosis. Furthermore, ZJS-178 impairs toxisome formation, leading to reduced production of the mycotoxin deoxynivalenol (DON). These findings position ZJS-178 as a promising antifungal agent that could pave the way for developing a novel fungicide to manage FHB and other *Fusarium*-related diseases.

**Supplementary Information:**

The online version contains supplementary material available at 10.1007/s44297-024-00034-z.

## Introduction

*Fusarium graminearum* is an aggressive fungal pathogen that causes Fusarium head blight (FHB) on multiple cereal crops worldwide [1]. Epidemics of FHB have directly caused huge yield losses of over millions of hectares in global wheat production areas [2]. In addition, *F. graminearum* produces various mycotoxins during infection of wheat, such as deoxynivalenol (DON) and zearalenone (ZEN), which may cause serious health impacts on humans and animals [3]. Due to the lack of wheat varieties with high resistance to *F. graminearum*, the application of chemical fungicides remains one of the most effective methods for managing FHB [4]. However, prolonged use of these chemical fungicides has led to resistance of *F. graminearum* against demethylation inhibitors (DMI) and benzimidazole (MBC) fungicides in fields [5, 6]. Moreover, the application of MBC or DMI at sub-lethal concentrations can stimulate mycotoxin biosynthesis, especially in the resistant strains [7]. Obviously, the lack of alternative chemical fungicides and the emergence of fungicide resistant *Fusarium* populations underscore the urgent need to develop novel antifungal compounds against FHB.

Phenamacril, a cyanoacrylate fungicide, developed by the Jiangsu Branch of the National Pesticide Research and Development Center of China, has been widely used over the past decade to control plant diseases caused by *Fusarium* spp., including FHB and rice bakanae disease [8, 9]. Interestingly, the antifungal activity of phenamacril is specific to some *Fusarium* species, such as *F. graminearum*, *F. asiaticum*, and *F. fujikuroi*, but it has low or no activity against other fungi [10, 11]. Previous studies have shown that phenamacril binds to *F. graminearum* myosin I (named FgMyoI hereafter), and inhibiting its ATPase activity [10]. More recently, phenamacril was found to bind to the actin-binding cleft in a new allosteric pocket of FgMyoI, which is collapsed in the structure of FgMyoI with a closed actin-binding cleft [11]. Myosin I, a single headed and membrane-associated protein, plays crucial roles in several fundamental cellular processes in fungal cells, including endocytosis and hyphal growth [12]. In addition, the hydrolysis of ATP by myosin I converts biological energy into mechanical force, facilitating the remodeling of the endoplasmic reticulum (ER) to form DON toxisomes, which are essential for DON production in *F. graminearum* [13]. As a result, phenamacril significantly reduces DON production by disrupting toxisome formation in vitro and in the field [13]. Currently, phenamacril is the only myosin I inhibitor used in agriculture, and its binding structure with FgMyoI has been elucidated [11]. This structural insight lays the groundwork for the development of novel compounds with enhanced antifungal activity targeting FgMyoI.

In this study, we optimized phenamacril derivatives and identified a highly active compound, ZJS-178. Exploring the mode action of ZJS-178 showed that ZJS-178 highly inhibits the activity of FgMyoI, and disrupts the formation of DON toxisome. Notably, we found that ZJS-178 also suppresses the internalization process of endocytosis in *F. graminearum*. This study underscores the potential for developing novel antifungal compounds that target FgMyoI to effectively combat FHB and other plant diseases caused by *Fusarium* species.

## Materials and methods

### Strains construction

The *F. graminearum* wild-type strain PH-1 (NRRL 31084) was used as a parental strain. Carbendazim-, tebuconazole- and pydiflumetofen-resistant strains were isolated from diseased wheat heads collected from the field, whereas phenamacril-resistant mutants were previously induced in the laboratory [10, 14].

To construct the FgTri1-GFP fusion cassette, the RP27 adaptor (5’-TTTCGTAGGAACCCAATCTTCAAA-3’) and open reading frame of FgTri1 without a stop codon were amplified as described previously [13]. The resulting PCR products were co-transformed with Xho1-digested pYF11 into the yeast strain XK1-25. The FgTri1-GFP vector was extracted using the yeast plasmid extraction kit (Solarbio, Beijing, China) and transferred into *E. coli* strain DH5α for amplification. After sequence verification, the vector was introduced into the wild-type PH-1 to generate PH-1::FgTri1-GFP.

### Synthesis of 2-cyanoacrylate compounds

The 2-cyanoacrylate compounds tested in this study were synthetized by Zhejiang Research Institute of Chemical Industry. For example, 0.80 g of the intermediate ZJS-178d (Fig. S1) and 25 mL of dichloromethane were added to a reaction flask. Then 1.8 mL of trifluoroacetic acid was added dropwise under an ice bath, and the mixture was stirred at room temperature overnight. After the reaction was complete, a saturated sodium bicarbonate aqueous solution was added, and the mixture was extracted twice with ethyl acetate. The combined organic phase was concentrated and separated by column chromatography using an eluent mixture of ethyl acetate and petroleum ether in a volume ratio of 1:1. This process yielded 0.54 g of the compound ZJS-178 with a yield of 92.2%.

### Fungicide susceptibility assay

To test fungicide susceptibility, 5 mm diameter mycelial plugs of each strain from the edge of a fresh colony were transferred onto potato dextrose agar (PDA) (200 g potato, 20 g glucose, 10 g agar and 1 L water) plates supplemented with each fungicide at the concentrations indicated in the figures. Each strain had three replicates per concentration were used. The plates were incubated at 25 °C for the time indicated in figure legends followed by measurement and imaging. Each experiment was repeated three times independently.

### Spore germination assay

The mycelia of the wild-type strain PH-1 were incubated in carboxymethyl cellulose (CMC) liquid medium (1 g NH_4_NO_3_, 1 g KH_2_PO_3,_ 0.5 g MgSO_4_·7H_2_O, 1 g yeast extract, 15 g CMC and 1 L water) at 25 °C, 150 rpm for 5 days to induce conidiation. The resulting conidia were then suspended in a 2% sucrose solution to obtain spore suspension at 10^4^ conidia per milliliter, which was added to a 12-well plate (3.0 mL per well). Each fungicide at indicated concentrations was then added to wells to assess its effects on conidial germination. After incubation at 25 °C for 5 h, the spores were stained with CFW dye and examined for germination under a confocal microscope. The experiment was repeated independently three times.

### Preparation of FgMyoI_IQ2_ and analysis of the ATPase activity

FgMyoI_IQ2_ (residues 1–757), a truncated FgMyoI containing the motor domain and two IQ motifs, was expressed in insect Sf9 cells and purified by anti-FLAG affinity chromatography [15]. The FgMyoI_IQ2_ M375A mutant was created by QuikChange site-directed mutagenesis with FgMyoI_IQ2_/pFastHFTc as the template and the recombinant baculovirus was prepared as described previously [10]. The actin-activated ATPase activity of FgMyoI_IQ2_ was measured in the presence of 40 μM actin and the indicated concentrations of phenamacril or ZJS-178 as described previously [15]. The experiment was repeated independently three times.

### Microscopic observation

The localization of the tagged proteins was observed with a Zeiss LSM780 confocal microscopy (Garl Zeiss AG, Jena, Germany). For toxisome observation, the FgTri1-GFP labeled strain was incubated in the trichothecene biosynthesis induction (TBI) medium (30 g sucrose, 1 g KH_2_PO_4_, 0.5 g MgSO_4_⋅7 H_2_O, 0.5 g KCl, 10 mg FeSO_4_⋅7H_2_O, 800 mg putrescine, and 200 μl trace element solution (5 g citric acid, 5 g ZnSO_4_⋅7 H_2_O, 0.25 g CuSO_4_⋅5 H_2_O, 50 mg MnSO_4_⋅H_2_O, 50 mg H_3_BO_3_, and 50 mg NaMoO_4_⋅2 H_2_O per 100 ml) at 28 °C for 24 h. Each fungicide was then added, and incubation continued for an additional 24 h before imaging. The experiment was repeated independently three times. Subcellular localization of FgUapC-GFP was carried out as previously described [16].

### Western blotting assay

The strain FgTri1-GFP was incubated in TBI medium at 28 °C, 150 rpm for 24 h. After incubation, each fungicide at indicated concentrations was then added and the incubation continued for an additional 24 h. Mycelia from each treatment were then harvested for protein extraction. Protein extraction was carried out as previously described [17], and the resulting proteins were separated by 12% sodium dodecyl sulfate–polyacrylamide gel electrophoresis (SDS-PAGE) and transferred to Immobilon-P transfer membrane (Millipore, Billerica, MA, USA). Immunoblot analysis was performed using the monoclonal anti-GFP ab32146 antibody (Abcam, Cambridge, UK) at 1:10000 dilution. The samples were also detected with the monoclonal anti-GAPDH antibody EM1101 (Hangzhou HuaAn Biotechnology Co., Ltd.) as a loading control. The intensity of immunoblot bands were quantified using the ImageQuantTL software. The experiment was independently repeated three times.

### DON production assay

To quantify DON production, PH-1 strain was grown in the toxin biosynthesis inducing (TBI) medium at 28 °C, 150 rpm for 24 h. Afterward, fungicides were added and the cultures were incubated for an additional 6 days. DON production of each sample was measured using the DON Quantification Kit Wis008 (Wise Science, Zhenjiang, China). The experiment was repeated independently three times.

## Results

### ZJS-178 highly inhibits mycelial growth and conidial germination of *F. graminearum*

Based on the structure of the crystal structures of phenamacril-bound FgMyoI, we designed and synthesized 101 derivatives (Table S1). Antifungal activity assays showed that 14 compounds exhibited higher antifungal activity than phenamacril, with ZJS-178 showing the highest activity against *F. graminearum* (Table S1).

**Fig. 1:**
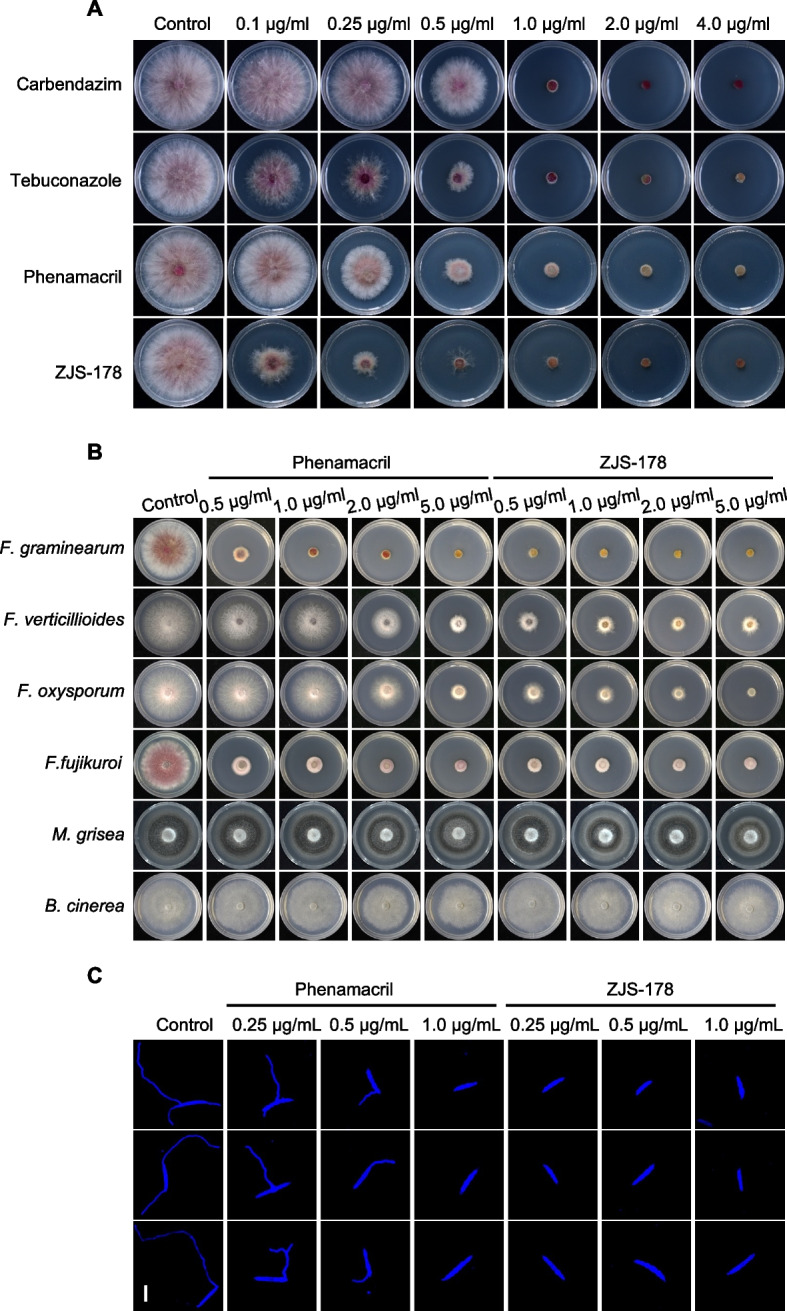
Antifungal activity of ZJS-178 against *F. graminearum* and other pathogenic fungi. **A** Sensitivity of *F. graminearum* to ZJS-178 and other fungicides. Plates were incubated at 25 °C for 2 days before imaging. **B** Antifungal activity of ZJS-178 and phenamacril against different pathogenic fungi. Plates were incubated at 25 °C for 2 days before imaging. **C** Inhibitory activity of ZJS-178 and phenamacril on conidial germination of *F. graminearum.* The effects of ZJS-178 and phenamacril on conidial germination were assessed after 5 h of incubation in 2% sucrose. Conidial germination inhibition was examined under a microscope, with images showing the extent of inhibition. Bar = 20 μm

To evaluate the antifungal efficacy of ZJS-178, we compared its sensitivity against *F. graminearum* with several commercial fungicides, including carbendazim (targeting beta-tubulin), tebuconazole (targeting 14-α-demethylase), and phenamacril (targeting FgMyoI). As shown in Fig. 1A, ZJS-178 demonstrated superior antifungal activity compared to the other tested fungicides. In addition, the EC_50_ values, determined via mycelial growth inhibition assay, were 0.361 μg/ml for phenamacril and 0.086 μg/ml for ZJS-178. These results indicate that ZJS-178 is more effective against *F. graminearum* than carbendazim, tebuconazole, and phenamacril.

Similar to phenamacril, which shows specificity to certain *Fusarium* species and minimal activity against other fungi [10], ZJS-178 exhibited specific antifungal activity against *Fusarium* spp., because it showed very low antifungal activity against *Magnaporthe grisea* and *Botrytis cinerea* (Fig. 1B). Compared to phenamacril, ZJS-178 was more effective against *Fusarium verticillioides* and *Fusarium oxysporum* (Fig. 1B). Notably, microscopic observation revealed that conidial germination was completely inhibited by ZJS-178 at a concentration of 0.25 μg/mL, without visible germ tubes. In contrast, conidia were still able to germinate in the presence of phenamacril at the same concentration (Fig. 1C).

### ZJS-178 doesn’t show cross-resistance with other groups of fungicides

**Fig. 2:**
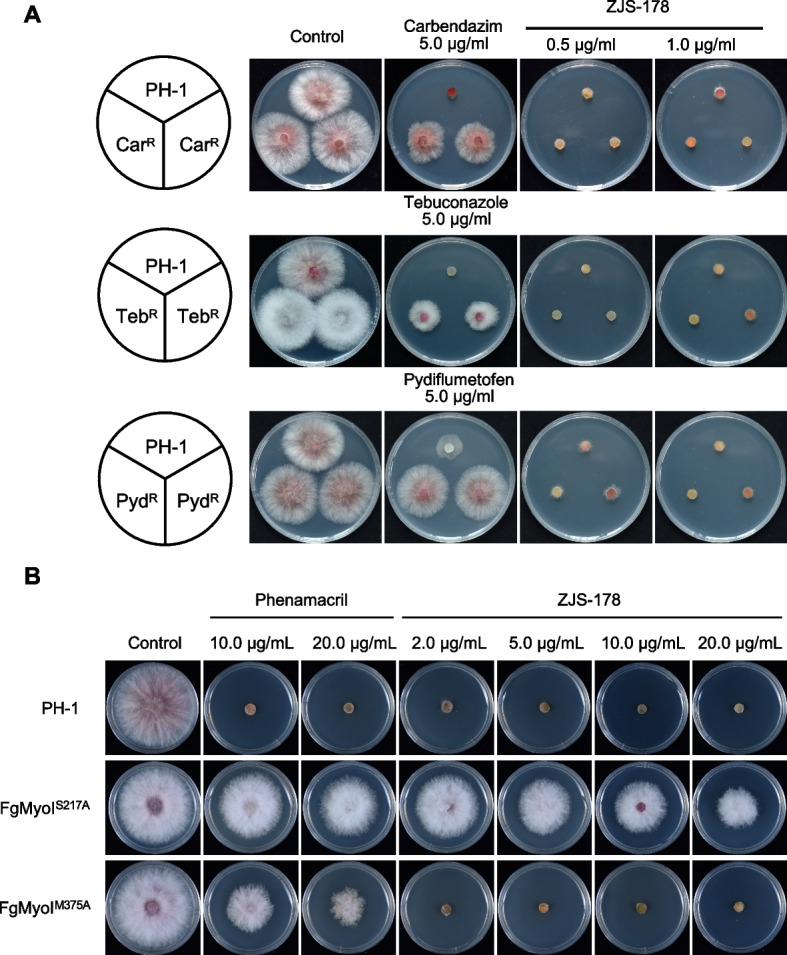
Determination of cross resistance between ZJS-178 and other groups of fungicides. **A** Antifungal activity of ZJS-178 against carbendazim-, tebuconazole-, and pydiflumetofen-resistant strains (Car^R^, Teb^R^, and Pyd^R^, respectively) of *F. graminearum*. Each strain was incubated on PDA plates supplemented with ZJS-178 or carbendazim, tebuconazole, and pydiflumetofen at indicated concentration in the figure. Plates were incubated at 25 °C for 2 days before imaging. **B** Antifungal activity of ZJS-178 against phenamacril-resistant mutants containing point mutations S217A and M375A in FgMyoI. Each strain was incubated on PDA plates supplemented with ZJS-178 or phenamacril at indicated concentration in the figure. Plates were incubated at 25 °C for 2 days before imaging

To assay cross-resistance between ZJS-178 and other fungicides, we tested the antifungal activity of ZJS-178 against *F. graminearum* strains resistant to commonly used fungicides including carbendazim, tebuconazole, and pydiflumetofen. As shown in Fig. 2A, while the wild-type PH-1 strain was completely inhibited by all tested fungicides, the field-resistant strains (Car^R^, Teb^R^, and Pyd^R^) were able to grow on media containing the respective fungicide. However, ZJS-178 effectively inhibited the mycelial growth of all these field-resistant strains, indicating no cross-resistance between ZJS-178 with other groups of fungicides.

Currently, phenamacril resistant isolates have not been detected in field, but laboratory-induced mutants with point mutations S217A and M375A in FgMyoI show moderate and high resistance to phenamacril, respectively [11]. Thus, we tested the sensitivity of ZJS-178 against these mutants, and found that the S217A mutant was resistant to both phenamacril and ZJS-178, while the M375A mutant was sensitive to ZJS-178 (Fig. 2B). These results indicate that ZJS-178 and phenamacril exhibit only partial cross-resistance.

### ZJS-178 inhibits ATPase activity of FgMyoI motor domain

**Fig. 3:**
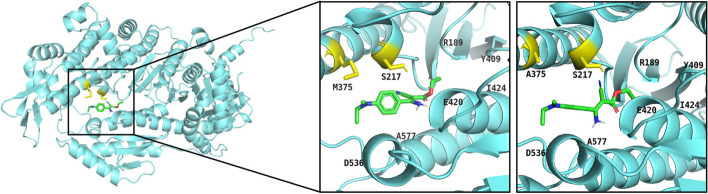
Molecular docking analysis of the interactions between ZJS-178 with FgMyoI or FgMyoI^M375A^

Myosin I proteins contain head (motor), neck (IQ) and tail domains. The motor domain includes an ATPase site and an ATP-sensitive F-actin-binding site, while the neck domain binds one or more copies of a single light chain [18]. To assess the effect of ZJS-178 on ATPase activity of FgMyoI, we overexpressed and purified the FgMyoI motor domain (FgMyoI-MIQ) from Sf9 cells via affinity chromatography. As shown in Table 1, ZJS-178 significantly inhibited the actin-activated ATPase activity of wild-type FgMyoI in a dose-dependent manner, with an IC_50_ of 0.035 μM, which is ~ 30 times lower than that of phenamacril (IC_50_ of 1.10 μM). The FgMyoI^S217A^ mutant displayed a moderate resistance to both phenamacril and ZJS-178 (~ 5 folds for both) (Table 1). Interestingly, the FgMyoI^M375A^ mutant was resistant to phenamacril, but virtually no resistant to ZJS-178 inhibition (Table 1). In addition, molecular docking showed that the binding energy of FgMyoI with ZJS-178 and phenamacril was -5.54 kcal/mol and -5.08 kcal/mol, respectively, indicating that ZJS-178 has a lower binding energy and stronger affinity for the target protein than phenamacril. Moreover, the N-ethyl-N-methylamino group of ZJS-178 was able to bind the Met375 or Ala375 of FgMyoI tightly (Fig. 3). These results confirm that ZJS-178 specifically targets wild-type FgMyoI and the FgMyoI^M375A^ mutant with high inhibitory activity, while showing reduced effectiveness against the FgMyoI^S217A^ mutant.

**Table 1:** Inhibition of phenamacril and ZJS-178 against ATPase activity of the wild-type and mutated FgMyoI

Myosin variants	IC_50_ of Phenamacril [μM]*	RF value**	IC_50_ of ZJS-178 [μM]*	RF value**
Wild type (WT)	1.10 ± 0.06***	/	0.035 ± 0.013	/
S217A	5.78 ± 0.11***	5.25***	0.190 ± 0.016	5.37
M375A	9.20 ± 2.08	8.36	0.0428 ± 0.0072	1.21

^*^All data are the mean ± std of three independent assays

^**^RF value = IC_50_ of FgMyoI mutant /IC_50_ of FgMyoI WT

^***^data from Ni et al.[15]

### ZJS-178 suppresses the endocytosis process

**Fig. 4:**
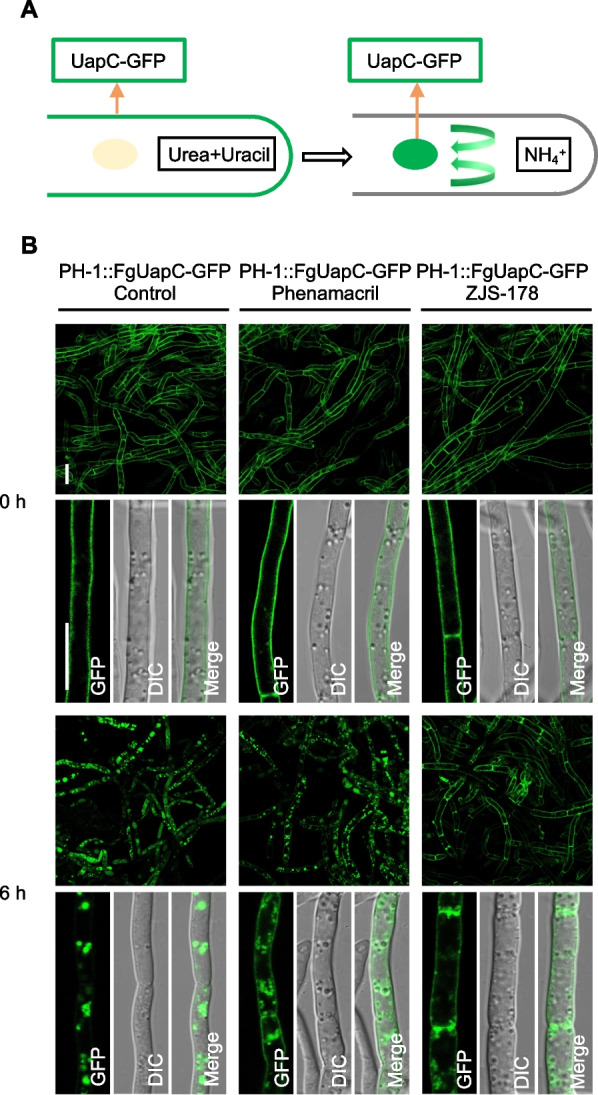
ZJS-178 inhibits endocytosis indicated by the internalization of FgUapC-GFP. **A** Schematic illustration of the FgUapC–GFP endocytosis. The strain bearing FgUapC–GFP was initially incubated on MM-N medium containing 5 mM urea and 0.05 mg/ml uracil as nitrogen sources. Under this condition, FgUapC–GFP was localized mainly on the plasma membrane. The strain was then transferred to ammonium medium condition for 6 h. This shift stopped the expression of FgUapC-GFP, leading to its endocytic delivery from the plasma membrane to the vacuole. **B** Fluorescence images of FgUapC-GFP endocytosis in the hyphae treated with ZJS-178 or phenamacril at the concentration of 1.0 μg/mL for 0 h and 6 h after the strain was shifted from urea and uracil to ammonium medium. Bar = 10 μm

Endocytosis is an energy-intensive process that requires overcoming significant osmotic pressure inside the cell [19]. Previous studies have shown that endocytosis not only depends on actin polymerization but also on myosin I to drive membrane invagination and scission in fungal cells [20, 21]. In this study, to observe potential effects of ZJS-178 on endocytosis, the plasma membrane-located protein FgFloA was labelled with GFP and introduced into PH-1, resulting in the strain PH::FgFloA-GFP. Using this strain, we found that ZJS-178 distinctly altered the plasma membrane morphology of *F. graminearum*, dramatically increasing the structures of resembling endocytic invaginations in the plasma membrane (Fig. S2). To further investigate impact of ZJS-178 on endocytosis, we used FgUapC, a uric acid-xanthine transporter and a well-established marker for analyzing endocytosis [16], labeled with GFP. This marker was introduced into the wild-type strain to create PH-1::FgUapC-GFP. As shown in Fig. 4A, B, treatment with 1.0 μg/mL of ZJS-178, and to a lesser extent with 1.0 μg/mL of phenamacril, resulted in FgUapC-GFP remaining predominantly at the plasma membrane and not entering the vacuole. In contrast, in the untreated control, FgUapC-GFP was primarily localized in the vacuole. These findings indicate that ZJS-178 strongly inhibits endocytosis of FgUapC-GFP in *F. graminearum*.

### ZJS-178 restrains toxisome formation and DON biosynthesis

**Fig. 5:**
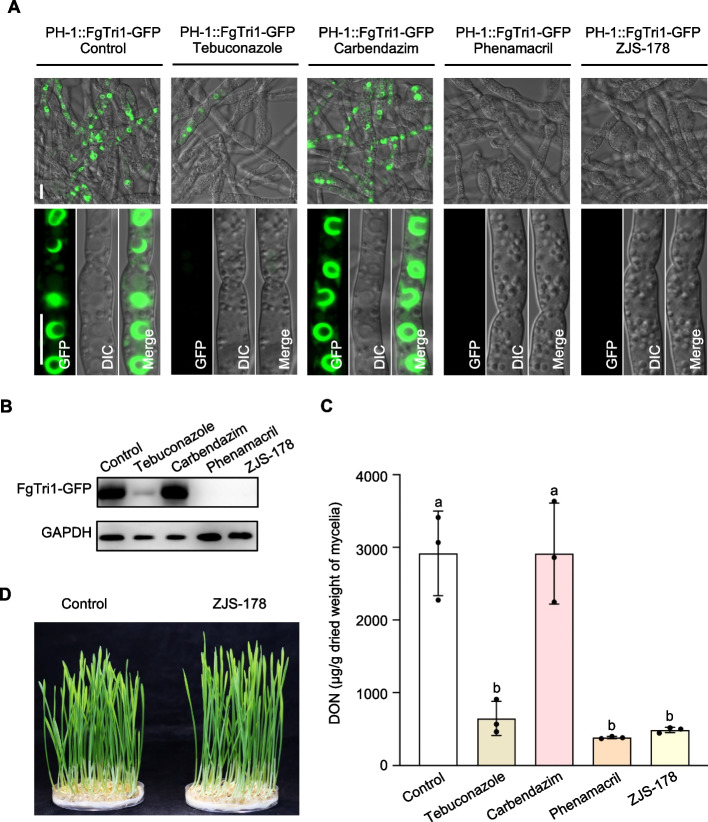
ZJS-178 disrupts DON toxisome formation and subsequently inhibits DON production. **A** Fluorescence images of PH-1::FgTri1-GFP growth in TBI at 28 °C for 24 h followed by treatment with 2.5 μg/ml tebuconazole, 1.4 μg/ml carbendazim, 1.0 μg/ml phenamacril or 1.0 μg/ml ZJS-178 for an additional 24 h, respectively. Bar = 10 μm. **B** Western blots of FgTri1-GFP protein isolated from the same set of samples used in 5A was detected with the anti-GFP antibody. The protein abundance of GPDA in each sample served as a loading control, detected with an anti-GAPDH antibody. **C** DON production of PH-1 treated with 2.5 μg/ml tebuconazole, 1.4 μg/ml carbendazim, 1.0 μg/mL phenamacril or 1.0 μg/ml ZJS-178, respectively, and cultured in TBI at 28 °C for 7 days. Different letters represent statistically significant differences according to a Fisher’s LSD test at *P* = 0.05. **D** Determination of toxicity of ZJS-178 to wheat seedlings. Wheat seeds were soaked in H_2_O supplemented with 20 μg/mL ZJS-178 at 25 °C for 24 h and then incubated for 7 days

DON poses a significant threat to human and animal health and acts as a virulence factor for *F. graminearum*. Thus, we investigated effect of ZJS-178 on DON toxisome formation and biosynthesis. We created a PH-1::Tri1-GFP strain by labeling GFP to the open reading frame of Tri1, which encodes the key enzyme for the final step in DON biosynthesis. This construct was introduced into the wild-type PH-1 strain to study toxisome formation as described previously [13, 22]. As shown in Fig. 5A, after the PH-1::Tri1-GFP strain was cultured in TBI medium at 28 °C for 48 h, Tri1-GFP was highly expressed and localized in spherical structures identified as DON toxisomes. However, in mycelia treated with 1.0 μg/mL ZJS-178, similar to phenamacril, the fluorescence signal of Tri1-GFP significantly diminished, and typical toxisomes were not observed. In contrast, 1.4 μg/mL carbendazim, a beta-tubulin inhibitor, did not affect toxisome formation (Fig. 5A). Additionally, the DMI fungicide tebuconazole at a high concentration (2.5 μg/mL) can partially inhibit the formation of toxisome (Fig. 5A). Immunoblot analysis using the anti-GFP antibody further confirmed that Tri1-GFP levels in the ZJS-178-treated PH-1::Tri1-GFP strain were nearly absent (Fig. 5B). Additionally, ZJS-178 at 1.0 μg/mL reduced DON biosynthesis by 83% compared to the control (Fig. 5C). These results suggest that ZJS-178 effectively inhibits DON production by interfering with toxisome formation in *F. graminearum*.

Regarding phytotoxicity, ZJS-178 did not affect the germination or growth of wheat seeds, even at a high concentration of 20 μg/mL (Fig. 5D). This indicates that ZJS-178 is likely safe for use on wheat crops.

## Discussion

Myosins are a superfamily of motor proteins that convert biological energy of ATP into mechanical energy, driving movement along actin filaments. In fungi, three myosin classes—Class I, II, and V—are essential for processes like cytokinesis, cell movement, and signal transduction [12]. Class I myosins, which are single-headed and membrane-associated, regulate membrane dynamics across nearly all eukaryotic cells [23]. In *Saccharomyces cerevisiae*, the closely related class I myosins Myo3 and Myo5 are critical for survival, with both deletions causing lethality or severely impaired growth [24, 25]. Filamentous fungi usually express a single myosin I isoform, such as MyoA in *Aspergillus nidulans*, essential for fungal growth [26]. Similarly, *F. graminearum* has a single MyoI protein, crucial for its growth development. This makes myosin I an attractive target for antifungal drugs, exemplified by phenamacril, which targets FgMyoI [10]. Interestingly, phenamacril, currently the sole myosin I inhibitor used in agriculture, shows high activity only against certain *Fusarium* species. Based on the high-resolution structure of the phenamacril-bound FgMyoI motor domain, we observed that other functional groups bind more tightly in the phenamacril binding pocket than the benzene ring [11]. This led us to focus on optimizing the benzene ring structure in our design of phenamacril derivatives in current study. As shown in Table S1, all 14 compounds with higher antifungal activity than phenamacril had modifications at the benzene ring's para-position. Among these, ZJS-178 exhibited the highest antifungal activity against *F. graminearum*. Molecular docking and myosin I ATPase assays confirmed that ZJS-178 has a stronger affinity for the target protein than phenamacril. These results suggest that based on structures of myosin I orthologues from other pathogenic fungi, the corresponding inhibitors could be developed.

**Fig. 6:**
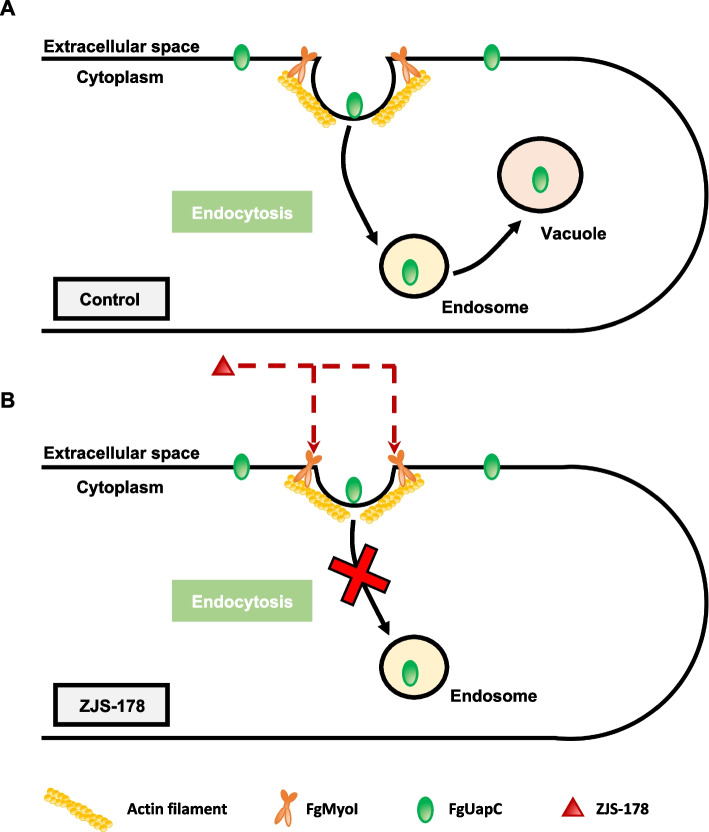
A proposed model for the role of FgUapC in endocytosis of *F. graminearum*. **A** In the control, FgUapC is internalized through endocytosis and subsequently transported to the vacuole for degradation. **B** ZJS-178 disrupts endocytosis by targeting FgMyoI, resulting in FgUapC remaining on the plasma membrane and preventing its internalization

Despite essential roles of myosin I in the survival of significant pathogenic fungi including *F. graminearum* and *Magnaporthe grisea*, the detailed functions of fungal myosin I orthologues remain largely unknown. In this study, we developed a species-specific myosin I inhibitor, which could be a valuable tool for studying myosin I functions in *Fusarium* species. For example, endocytosis, a critical process where cells internalize extracellular materials via endocytic vesicles, involves stages of invagination, elongation, and vesicle formation [12]. This process requires overcoming high intracellular osmotic pressure. While actin polymerization was once thought to be the primary driver [27], recent studies suggest that myosin I also plays a role by pushing the actin network from the plasma membrane to the cytoplasm, facilitating actin monomer incorporation [12, 21]. Our study demonstrates that ZJS-178 significantly impairs endocytosis, underscoring the crucial role of myosin I in this process (Fig. 6).

Trichothecenes are a large family of toxic sesquiterpenoid metabolites produced by certain *Fusarium* species. *F. graminearum* produces several trichothecenes, including DON, nivalenol (NIV), and acetylated derivatives 15-ADON and 3-ADON. The biosynthesis of these toxins involves 15 *Tri* genes located on three chromosomes in *F. graminearum* [28]. Efficient DON production requires the subcellular compartmentalization of biosynthetic enzymes like Tri4 and Tri1 into spherical organelle known as "toxisome", which is highly organized structure derived from the smooth endoplasmic reticulum [29, 30]. A previous study showed that the FgMyoI-actin cytoskeleton provides the mechanical energy needed for ER remodeling to form toxisomes in *F. graminearum* [13]. Disruption of FgMyoI and actin hinders toxisome formation, leading to a significant reduction in DON accumulation [13]. Our study further shows that ZJS-178 is able to dramatically inhibit toxisome formation, thereby blocking DON biosynthesis by targeting FgMyoI, indicating its high potential for managing DON contamination in wheat.

Despite extensive efforts to develop specific myosin inhibitors, few small molecules meet the functional and specificity criteria for practical use [10]. In agriculture, phenamacril has been commercialized as a myosin I inhibitor. Our study optimized phenamacril's structure, yielding ZJS-178, which exhibits superior antifungal activity against *F. graminearum* compared to phenamacril. Notably, ZJS-178 effectively combats phenamacril-resistant strains with the M375A mutation in FgMyoI and shows enhanced activity against other *Fusarium* species, including *F. verticillioides* and *F. oxysporum*. Additionally, ZJS-178 remains effective against field strains resistant to carbendazim, tebuconazole, and pydiflumetofen, indicating its potential as a novel fungicide for *Fusarium* diseases of various crops.

## Supplementary Information

Additional file 1: Figure S1. Synthesis of the 2-cyanoacrylate compound ZJS-178.

Additional file 2: Figure S2. Impacts of ZJS-178 on the plasma membrane were observed using FgFloA-GFP and the membrane dye FM4-64.

Additional file 3: Table S1. Structures and antifungal activities against *F. graminearum* of 101 phenamacril derivates (2-cyanoacrylate compounds) tested in this study.

## Data Availability

All data supporting the finding of this study are available within the paper and its Supplementary Information.
