# Supplementary figures and images for: A novel highly antifungal compound ZJS-178 targeting myosin I inhibits the endocytosis and mycotoxin biosynthesis of Fusarium graminearum
Source: Crop Health. 2024 Sep 26;2(1):14. doi: 10.1007/s44297-024-00034-z (PMC12825925; doi:10.1007/s44297-024-00034-z)

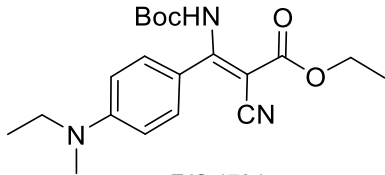

ZJS-178d

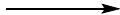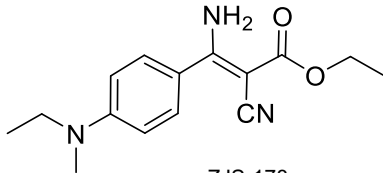

ZJS-178

Supplement: Supplementary file 1 — Additional file 1: Figure S1. Synthesis of the 2-cyanoacrylate compound ZJS-178. [file 44297_2024_34_MOESM1_ESM.pdf]

5 h

PH-1::FgFloA-GFP  
Control

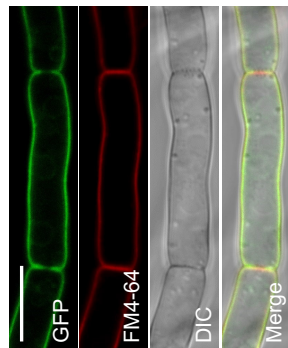

PH-1::FgFloA-GFP  
Phenamacril 1.0  $\mu\text{g/mL}$

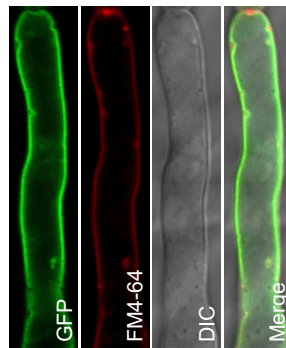

PH-1::FgFloA-GFP  
ZJS-178 1.0  $\mu\text{g/mL}$

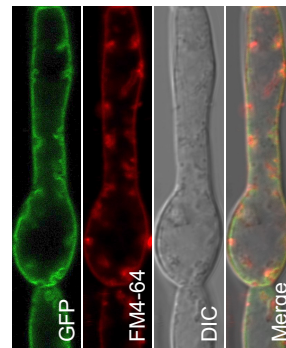

Supplement: Supplementary file 2 — Additional file 2: Figure S2. Impacts of ZJS-178 on the plasma membrane were observed using FgFloA-GFP and the membrane dye FM4-64. [file 44297_2024_34_MOESM2_ESM.pdf]
