# Supplementary material for: A novel highly antifungal compound ZJS-178 targeting myosin I inhibits the endocytosis and mycotoxin biosynthesis of Fusarium graminearum
Source: Crop Health. 2024 Sep 26;2(1):14. doi: 10.1007/s44297-024-00034-z (PMC12825925; doi:10.1007/s44297-024-00034-z)
Supplement: Supplementary file 3 — Additional file 3: Table S1. Structures and antifungal activities against F. graminearum of 101 phenamacril derivates (2-cyanoacrylate compounds) tested in this study. [file 44297_2024_34_MOESM3_ESM.docx]

| **Table S1.** Structures and antifungal activities against *F. graminearum* of 101 phenamacril derivates (2-cyanoacrylate compounds) tested in this study. | | | | |
| --- | --- | --- | --- | --- |
| **Number** | **Compound structure** | | **Activity rating*** | **EC_50_ (μg/ml)** |
| ZJS-089 | 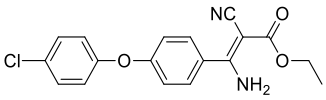 | **–** | | / |
| ZJS-090 | 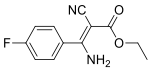 | **–** | | / |
| ZJS-091 | 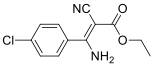 | **–** | | / |
| ZJS-098 | 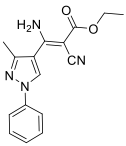 | **–** | | / |
| ZJS-106 | 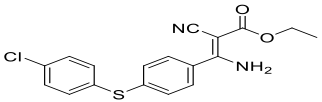 | **–** | | / |
| ZJS-107 | 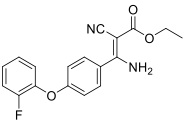 | **–** | | / |
| ZJS-108 | 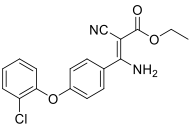 | **–** | | / |
| ZJS-109 | 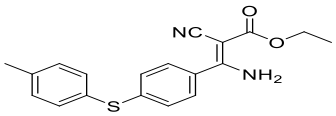 | **–** | | / |
| ZJS-110 | 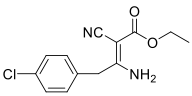 | **–** | | / |
| ZJS-111 | 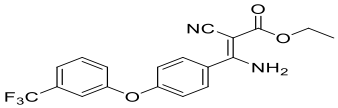 | **–** | | / |
| ZJS-114 | 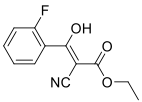 | **–** | | / |
| ZJS-115 | 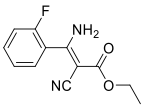 | **–** | | / |
| ZJS-116 | 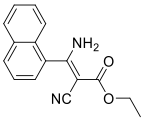 | **–** | | / |
| ZJS-117 | 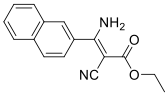 | **+** | | / |
| ZJS-118 | 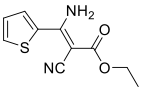 | **+** | | / |
| ZJS-119 | 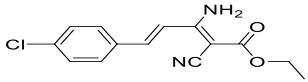 | **–** | | / |
| ZJS-120 | 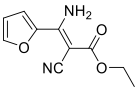 | **–** | | / |
| ZJS-121 | 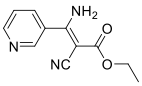 | **–** | | / |
| ZJS-122 | 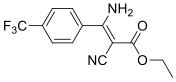 | **–** | | / |
| ZJS-123 | 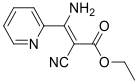 | **–** | | / |
| ZJS-124 | 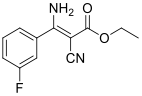 | **+** | | / |
| ZJS-125 | 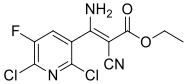 | **–** | | / |
| ZJS-126 | 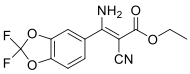 | **–** | | / |
| ZJS-127 | 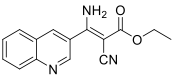 | **–** | | / |
| ZJS-128 | 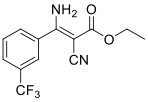 | **–** | | / |
| ZJS-129 | 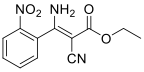 | **–** | | / |
| ZJS-130 | 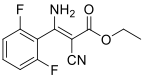 | **–** | | / |
| ZJS-131 | 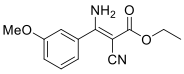 | **–** | | / |
| ZJS-132 | 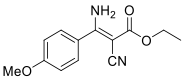 | **++** | | 0.181 |
| ZJS-133 | 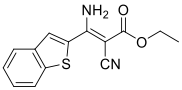 | **+** | | / |
| ZJS-134 | 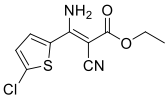 | **–** | | / |
| ZJS-141 | 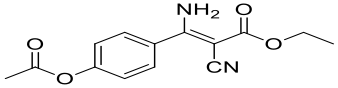 | **–** | | / |
| ZJS-142 | 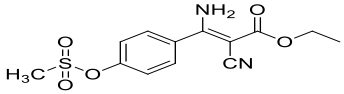 | **–** | | / |
| ZJS-143 | 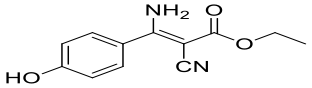 | **–** | | / |
| ZJS-144 | 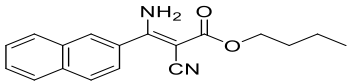 | **–** | | / |
| ZJS-145 | 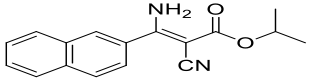 | **–** | | / |
| ZJS-146 | 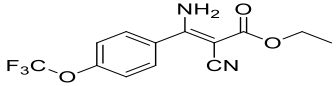 | **–** | | / |
| ZJS-147 | 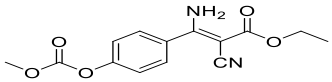 | **–** | | / |
| ZJS-148 | 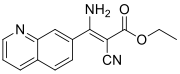 | **–** | | / |
| ZJS-150 | 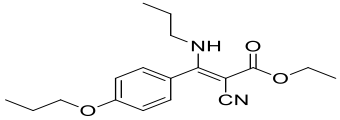 | **–** | | / |
| ZJS-151 | 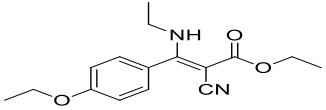 | **–** | | / |
| ZJS-152 | 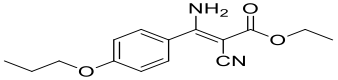 | **–** | | / |
| ZJS-153 | 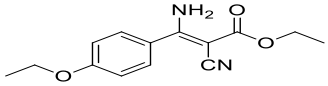 | **+** | | / |
| ZJS-154 | 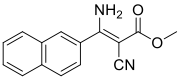 | **–** | | / |
| ZJS-155 | 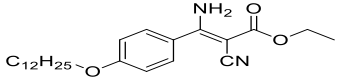 | **–** | | / |
| ZJS-156 | 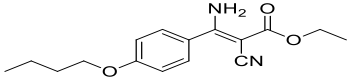 | **–** | | / |
| ZJS-157 | 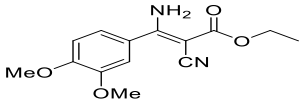 | **–** | | / |
| ZJS-158 | 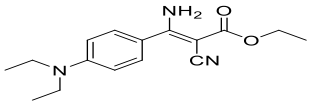 | **++** | | 0.093 |
| ZJS-159 | 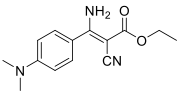 | **++** | | 0.124 |
| ZJS-160 | 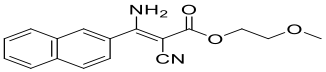 | **–** | | / |
| ZJS-161 | 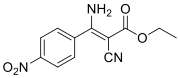 | **–** | | / |
| ZJS-162 | 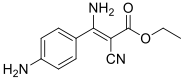 | **–** | | / |
| ZJS-163 | 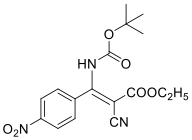 | **–** | | / |
| ZJS-164 | 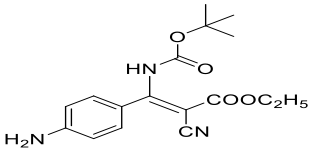 | **–** | | / |
| ZJS-165 | 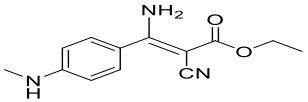 | **++** | | 0.358 |
| ZJS-166 | 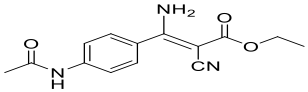 | **–** | | / |
| ZJS-167 | 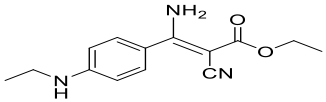 | **++** | | 0.320 |
| ZJS-168 | 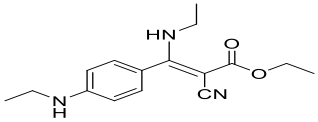 | **–** | | / |
| ZJS-169 | 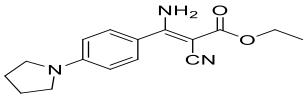 | **+** | | / |
| ZJS-170 | 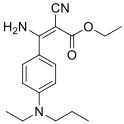 | **++** | | 0.318 |
| ZJS-171 | 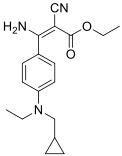 | **+** | | / |
| ZJS-172 | 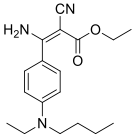 | **–** | | / |
| ZJS-173 | 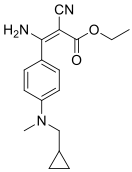 | **+** | | / |
| ZJS-174 | 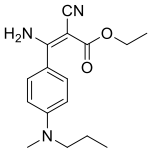 | **++** | | 0.231 |
| ZJS-175 | 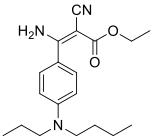 | **–** | | / |
| ZJS-176 | 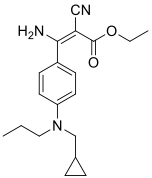 | **–** | | / |
| ZJS-177 | 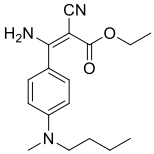 | **–** | | / |
| ZJS-178 | 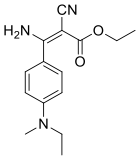 | **++** | | 0.086 |
| ZJS-179 | 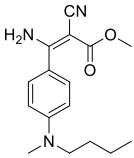 | **–** | | / |
| ZJS-180 | 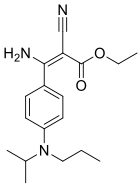 | **–** | | / |
| ZJS-181 | 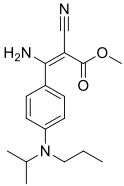 | **–** | | / |
| ZJS-182 | 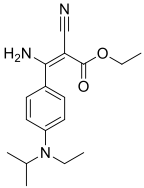 | **–** | | / |
| ZJS-183 | 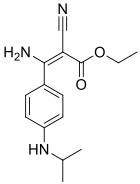 | **++** | | 0.233 |
| ZJS-184 | 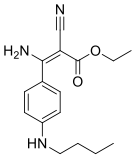 | **+** | | / |
| ZJS-185 | 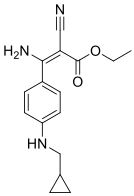 | **+** | | / |
| ZJS-186 | 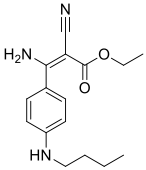 | **–** | | / |
| ZJS-187 | 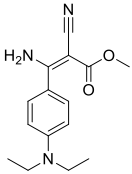 | **–** | | / |
| ZJS-188 | 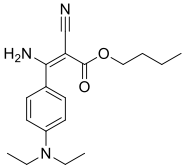 | **–** | | / |
| ZJS-189 | 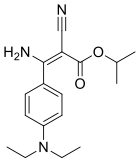 | **–** | | / |
| ZJS-190 | 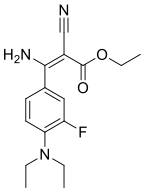 | **++** | | 0.240 |
| ZJS-191 | 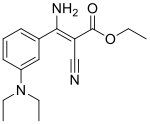 | **–** | | / |
| ZJS-192 | 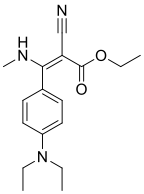 | **–** | | / |
| ZJS-193 | 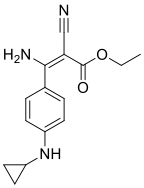 | **+** | | / |
| ZJS-194 | 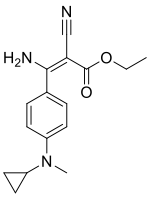 | **++** | | 0.258 |
| ZJS-195 | 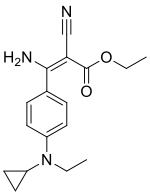 | **+** | | / |
| ZJS-196 | 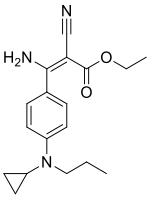 | **–** | | / |
| ZJS-197 | 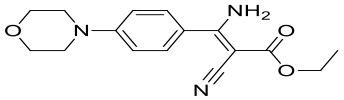 | **–** | | / |
| ZJS-198 | 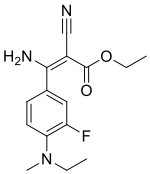 | **++** | | 0.131 |
| ZJS-199 | 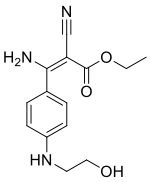 | **–** | | / |
| ZJS-200 | 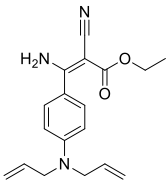 | **–** | | / |
| ZJS-201 | 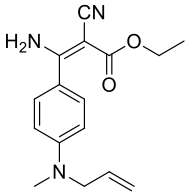 | **+** | | / |
| ZJS-202 | 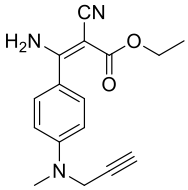 | **++** | | 0.180 |
| ZJS-203 | 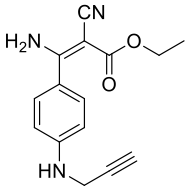 | **+** | | / |
| ZJS-204 | 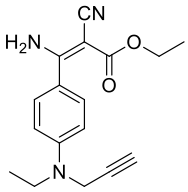 | **++** | | 0.164 |
| ZJS-205 | 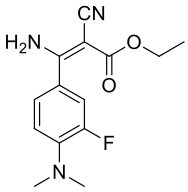 | **+** | | / |
| ZJS-206 | 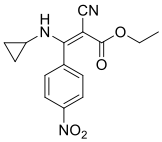 | **–** | | / |
| ZJS-207 | 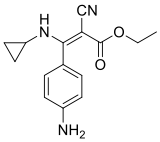 | **–** | | / |
| ZJS-208 | 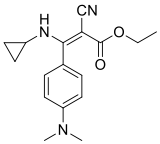 | **–** | | / |
| ZJS-209 | 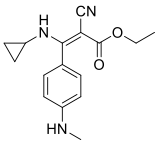 | **–** | | / |
| ZJS-210 | 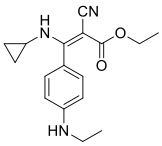 | **–** | | / |
| ZJS-211 |  | **–** | | / |

* The activity of the compound is lower than phenamacril (**–**); + and ++ indicate that the activity of the compound is similar and higher than phenamacril, respectively.
